# Modulation of recognition memory performance by light and its relationship with cortical EEG theta and gamma activities

**DOI:** 10.1016/j.bcp.2020.114404

**Published:** 2021-09

**Authors:** Sibah Hasan, Shu K.E. Tam, Russell G. Foster, Vladyslav V. Vyazovskiy, David M. Bannerman, Stuart N. Peirson

**Affiliations:** a Sleep and Circadian Neuroscience Institute (SCNi), Nuffield Department of Clinical Neurosciences, University of Oxford, United Kingdom; b Neuro-Bio Ltd., Abingdon, United Kingdom; c Department of Physiology, Anatomy and Genetics, University of Oxford, United Kingdom; d Behavioural Neuroscience Unit, Department of Experimental Psychology, University of Oxford, United Kingdom

**Keywords:** Light intensity, Recognition memory performance, EEG theta, EEG gamma, Mice

## Abstract

Acute exposure to light exerts widespread effects on physiology, in addition to its key role in photoentrainment. Although the modulatory effect of light on physiological arousal is well demonstrated in mice, its effect on memory performance is inconclusive, as the direction of the effect depends on the nature of the behavioural task employed and/or the type of stimulus utilised. Moreover, in all rodent studies that reported significant effects of light on performance, brain activity was not assessed during the task and thus it is unclear how brain activity was modulated by light or the exact relationship between light-modulated brain activity and performance. Here we examine the modulatory effects of light of varying intensities on recognition memory performance and frontoparietal waking electroencephalography (EEG) in mice using the spontaneous recognition memory task. We report a light-intensity-dependent disruptive effect on recognition memory performance at the group level, but inspection of individual-level data indicates that light-intensity-dependent facilitation is observed in some cases. Using linear mixed-effects models, we then demonstrate that EEG fast theta (*θ*) activity at the time of encoding negatively predicts recognition memory performance, whereas slow gamma (*γ*) activity at the time of retrieval positively predicts performance. These relationships between *θ*/*γ* activity and performance are strengthened by increasing light intensity. Thus, light modulates *θ* and *γ* band activities involved in attentional and mnemonic processes, thereby affecting recognition memory performance. However, extraneous factors including the phase of the internal clock at which light is presented and homeostatic sleep pressure may determine how photic input is translated into behavioural performance.

## Graphical abstract

**Figure:**
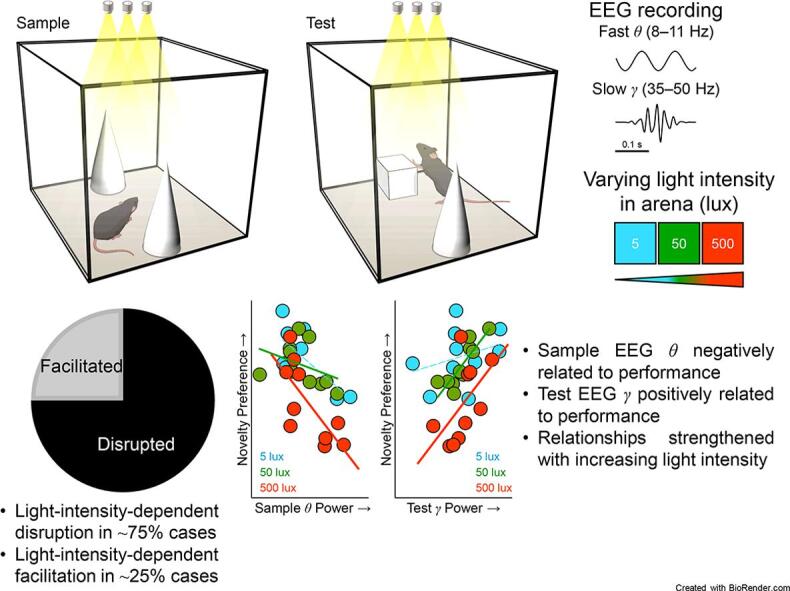

## 1 Introduction

Light exerts rapid and widespread effects on physiology and behaviour, in addition to its key role in circadian entrainment. There is now considerable evidence from human studies that bright light influences body temperature, subjective measures of sleepiness, arousal/alertness (measured by waking electroencephalography; EEG), thereby affecting cognitive performance [1], [2], [3], [4]. For example, in numerous key human studies, it has been reported that light exposure leads to decreased waking EEG theta (*θ*) and alpha (*α*) oscillatory activities in 5–9 Hz frequency bands, as well as decreased slow-wave *δ* activity (<5 Hz) associated with sleep pressure [4], [5], [6], [7], [8], [9], [10], [11]. In addition, neuroimaging studies have revealed that bright light can activate the hypothalamus, thalamus, brain stem, and limbic areas, eventually leading to widespread activation of different cortical areas [12], [13], [14], [15], [16].

Although mice are nocturnal, their response to light across the circadian cycle - as described by the phase-response curve - is broadly similar to that in humans [17]. In mice, acute exposure to bright light elevates heat rate, locomotor activity [18], and plasma and brain corticosterone levels [19]. This indicates that, like humans, bright light increases arousal/alertness in mice. Repeated exposure to abnormal lighting, such as non-24 h photoperiods, can have negative effects on cardiovascular physiology and mood-related behaviour in mice [20], [21]. Although effects of light on physiological arousal are well demonstrated, its effects on learning and memory performance are unclear. For example, light enhances unconditioned startle responses to loud auditory stimuli [22], as well as Pavlovian conditioned freezing responses to stimuli that predict occurrence of foot shocks [23], [24]. By contrast, in non-aversive behavioural paradigms performance can be disrupted under bright light [25], [26]. Unlike the key human studies, however, brain activity was not assessed in these studies and thus we do not know if mouse brain activity was modulated by light in a manner analogous to that in humans. This limits our understanding of the mechanism by which light modulates arousal/alertness and performance in the mammalian brain.

**Fig. 1:**
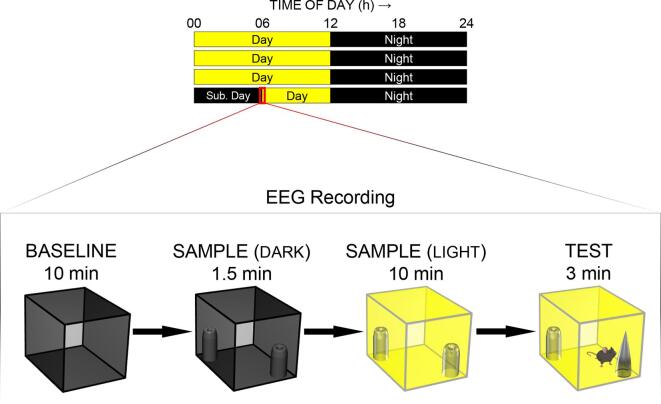
The spontaneous recognition memory task. Five telemetry-implanted mice were housed under a 12 h:12 h light–dark cycle prior to EEG recording and behavioural testing. On each recording day, mice were kept in complete darkness in their home cages from circadian time (CT) 00 to 06 h. EEG and EMG recordings and behavioural testing were started at CT 06 h. Each object or odour trial began with a 10-min BASELINE period, during which the mouse explored the empty arena in complete darkness. The animal was removed from the arena at the end of BASLINE and returned to its home cage for 1 min. It was then put back into the dark arena, now containing two replicates of an identical object or two identical shot glasses with the same odour cue, and the animal was allowed to explore the samples for 90 s (DARK SAMPLE). A light pulse of 5, 50, or 500 lx, was then presented immediately after the 90-s DARK SAMPLE period, and the mouse was allowed to explore the same objects or odours for another 10 min in the illuminated arena (LIGHT SAMPLE). During the 3-min delay where the mouse was returned to its home cage, one replicate of the sample object or odour was replaced by a novel object or odour cue, and the mouse was allowed to explore the novel and familiar objects or odours for 3 min during the TEST period.

Here we examine the effect of light of varying intensities, 5, 50, and 500 lx, on performance and frontoparietal waking EEG in mice using the spontaneous recognition memory task [27], [28], [29]. The task assesses the mouse’s innate preference for novel over familiar stimuli, and this novelty preference provides a measure of recognition memory sensitivity in rodents and humans [30]. Object and odour cues are used to see if the effect of light differs between different stimulus modalities. We examine EEG fast *θ* (8.5–11.2 Hz) and slow gamma (*γ*: 35–50 Hz) activities at the time of stimulus encoding during the sample phase, as well as at the time of memory retrieval during the test phase (Fig. 1). This allows us to assess how light-modulated *θ* and *γ* activities are related to recognition memory performance. As *θ* and *γ* signals originated primarily from the septohippocampal system and interconnected cortical areas are linked to various waking behaviours, such as locomotion, exploration, stimulus sampling, as well as attentional and mnemonic processes [31], [32], [33], [34], [35], it is anticipated that light of varying intensities would modulate EEG *θ* and *γ* signals to different extents, leading to different levels of recognition memory performance.

## 2 Materials and methods

### 2.1 Animals and lighting

A total of 17 C57BL/6JOlaHsd male mice aged 10–13 weeks old were purchased from Envigo (Bicester, England); 5 mice received telemetric implantation for EEG recording under recognition testing, whereas the rest of the animals were not implanted and only received recognition testing. Animals were singly housed immediately upon arrival at the colony and were given food and water *ad libitum* for the duration of the entire experiment. Their cages were placed within a light-tight and sound-attenuated chamber with a 12 h:12 h light–dark cycle, with a light level of 50 lx in the light phase provided by cool white LEDs [36]. The same type of cool white LEDs (Luxeon Star LEDs; Quadica Developments, Lethbridge, Canada) was used for housing and in all experiments. The spectral power distribution of our cool white LEDs was bimodal, comprising a higher and narrower peak at ~460 nm and a lower and broader peak at ~560 nm [26]. The temperature of the colony where the light-tight chamber was located was maintained at 22 ± 1 °C. All experimental procedures were carried out in accordance with the United Kingdom Animals (Scientific Procedures) Act 1986 under Project Licence 30/2812 and Personal Licence I459D3D59.

### 2.2 Surgery

Five mice received telemetric implantation at the age of 16 weeks. Each mouse was anaesthetised with isoflurane (IsoFlo; Abbott Laboratories, Maidenhead, England; 4.5% induction and 0.7–2.25% maintenance), and a 3.9-g telemetric transmitter with a volume of 1.9 cm^3^ (TL11M2-F20-EET; Data Sciences International, New Brighton, Minnesota) was implanted intraperitoneally as described in [37]. An incision was made along the midline of the scalp, and the stainless steel wires from the telemetric transmitter, which were protected by silicone tubing, were passed subcutaneously along the neck and exited through the incision. Two stainless-steel EEG electrodes (length of screw shaft: 2.4 mm; outer diameter of screw thread: 1.19 mm) were implanted epidurally over the right frontal and parietal cortices as described in [38]. The stainless steel wires from the telemetric transmitter were trimmed and connected to the EEG electrodes, which were anchored with dental cement (RelyX Arc; Kent Express, Kent, England). Two electromyography (EMG) stainless-steel leads were inserted approximately 5 mm apart from each other into the neck muscles and were sutured in place. Analgesics including buprenorphine (Vetergesic; Sogeval, York, England; 0.01 mg kg^−1^) and meloxicam (Metacam; Boehringer Ingelheim, Bracknell, England; 0.5 mg kg^−1^) were administered subcutaneously prior to surgery, and 0.5 mL saline was administered subcutaneously immediately after surgery to prevent dehydration. A second dose of meloxicam (0.5 mg kg^−1^) was given the next day.

### 2.3 Procedure

For the 5 implanted mice, the experiment began thirteen weeks after surgery. Each mouse was first given two 10-min habituation sessions in the 20 cm × 20 cm × 20 cm white acrylic arena at Zeitgeber Time (ZT) 6 on two separate days; the arena did not contain any object and was *not* illuminated during these habituation trials. After the habituation phase, the mouse received three object recognition trials and three odour recognition trials (1 trial per day; Fig. 1). At the start of each recording day, LED lights in the light-tight chamber were not turned on at ZT 0 and it remained dark for 6 h, similar to that in [18]. The telemetric transmitter was activated, and EEG and EMG recordings were started, in the middle of the subjective light phase [i.e. Circadian Time (CT) 6].

#### 2.3.1 Implanted animals’ recording days 1–3: object recognition trials

Each of the three object recognition trials consisted of: (*a*) a 10-min baseline period (BASELINE), during which the mouse was allowed to explore the empty arena in darkness; (*b*) a 90-s object pre-exposure period (DARK SAMPLE), during which the mouse was allowed to explore two replicates of the sample object on the left and right sides of the arena in complete darkness; (*c*) a 10-min sample phase (LIGHT SAMPLE), during which a light pulse of either 5, 50, or 500 lx was given while the mouse continued to explore and retain the sample objects; (*d*) a 3-min delay period, during which the mouse was removed from the arena, and one replicate of the sample object was replaced by an entirely novel object and the other replicate of the sample object was replaced by a third replicate of the same object that had not been presented to the mouse; and finally, (*e*) a 3-min test period (TEST), during which the mouse was returned to the illuminated arena (with the same light intensity as in the sample phase), and was allowed to explore the novel and familiar objects, including encoding of the new object (Fig. 1). The two types of object differed in various sensory modalities and could be differentiated based on visual as well as non-visual cues; a detailed description of object used can be found in our previous report [26]. The identities of the novel and familiar objects, as well as their locations in the arena at test, were counterbalanced across the 5-lux, 50-lux, and 500-lux conditions.

#### 2.3.2 Implanted animals’ recording days 4–6: odour recognition trials

After five days of re-entrainment to the original 12 h:12 h light–dark cycle, animals received three odour recognition trials (1 trial per day) at CT 6. Various essential oils, including lemon, vanilla, and peppermint extracts (Dr. Oetker, Bielefeld, Germany), orange, chocolate, and rose extracts (Nielsen-Massey, Leeuwarden, Netherlands), as well as banana and jasmine essence (Double Seahorse, Bangkok, Thailand), were used as odour cues. Shortly prior to each recording trial, 0.5 mL of each stimulus was delivered with a syringe into a clear shot glass with a base diameter of 4.6 cm and a height of 6.4 cm. There were eight identical shot glasses, so that different glasses could be presented in sample and test phases. Similar to the object trials, each of the three odour recognition trials consisted of: (*a*) a 10-min baseline period in the empty arena (BASELINE); (*b*) a 90-s odour pre-exposure period (DARK SAMPLE), during which the mouse was allowed to explore the two shot glasses containing the sample odour cue in complete darkness; (*c*) a 10-min sample phase (LIGHT SAMPLE), during which a light pulse of either 5, 50, or 500 lx was given while the mouse continued to explore the sample odour cues; (*d*) a 3-min delay period, during which the mouse was removed from the arena, and a novel odour cue was introduced into the arena; and finally, (*e*) a 3-min test period (TEST), during which the mouse was returned to the illuminated arena and was allowed to explore the novel and familiar odour cues. The light level during the test phase remained at 50 lx across all odour trials. All other aspects of the odour trials were identical to the object trials.

#### 2.3.3 Non-implanted animals

The behavioural procedure for the object recognition testing in non-implanted mice was similar to that used in implanted mice, except that: (*a*) there was no BASELINE or DARK SAMPLE period on each trial; (*b*) and light levels during sample and test phases were 10 lx and 350 lx. Each mouse received two object recognition trials; for half of the animals 10 lx was used on the first trial and 350 lx on the second trial, whereas this arrangement was reversed for the rest of the animals. All other procedural details remained identical.

### 2.4 EEG data acquisition and processing

For the 5 implanted mice, on each recording trial telemetric EEG and EMG data were transmitted at 455 kHz to an RPC-1 receiver (Data Sciences International, New Brighton, Minnesota). These signals were sampled at 250 Hz, recorded using the Dataquest ART system (Data Sciences International, New Brighton, Minnesota), and were high-pass (3 dB, 1.0 Hz) and low-pass antialiasing (49.83 Hz) analogue filtered. Artefacts in the waking EEG were identified by visual inspection of EEG signals displayed on a computer screen for consecutive 4-s epochs; these were excluded from subsequent spectral analyses. EEG power spectra were computed for consecutive 4-s epochs using a fast Fourier transform (FFT) with the Hanning window (frequency range: 0.98–49.83 Hz; resolution: ~0.25 Hz). For each recording trial, EEG spectra (in μV^2^ per 0.25 Hz) from 2.20 Hz to 49.83 Hz during sample and test phases were expressed as a percentage of the EEG spectrum (in μV^2^ per 0.25 Hz) in the corresponding frequency bins during the preceding baseline period. Data in the low-frequency range (0.98–1.95 Hz) were excluded due to movement artefacts. Spectral changes in the waking EEG during sample and test phases were divided into multiple time bins, such that each bin contained an equal number of 4-s waking epochs. To examine the effect of light intensity on brain activity related to recognition memory performance, we focused on fast *θ* (8.5–11.2 Hz) and slow *γ* frequency bands (35–50 Hz), as these signals are related to learning and memory performance in different behavioural tasks in rodents [31], [32], [33], [34], [35].

### 2.5 Statistical analyses

#### 2.5.1 Classical parametric tests

For the implanted mice, Light Intensity (5, 50, or 500 lx) × Stimulus Type (Objects or Odours) within-subjects analyses of variance (ANOVAs) were conducted on recognition scores, stimulus exploration times (s min^−1^), and distance travelled (m min^−1^). In addition, multiple one-sample *t* tests were used to compare mean recognition ratio scores against the value of 0, which indicates that mice did not discriminate between novel and familiar stimuli. Similar ANOVAs and one-sample *t* tests were conducted for the non-implanted mice. These analyses were conducted in SPSS (IBM). *α* = 0.05 was adopted for all parametric analyses.

#### 2.5.2 Linear mixed-effects models

For the implanted mice, relationships between EEG *θ*/*γ* power during stimulus encoding/memory retrieval and recognition memory performance were assessed using linear mixed-effects models, with *θ*/*γ* Power as well as Light Intensity × *θ*/*γ* Power interaction as fixed-effects predictors; individual animals and stimulus types were entered as random effects i.e. fixed slopes but varying intercepts among individual mice and in the two types of trial. Similar linear mixed-effects models were also conducted with stimulus exploration times (s min^−1^) and distance travelled (m min^−1^) as response variables. The significance of a fixed-effect predictor was assessed using the jackknife method, a conservative approach that assesses bias of any influential data point in the model [39]. As there were 30 data points in total (5 mice × 3 light intensities × 2 types of stimulus), to examine the significance of each predictor 30 pairs of linear mixed-effects models - models with *versus* without the predictor of interest - were conducted. Each time one data point was left out sequentially. Significance was determined by examining the change in deviance (−2 × maximum log-likelihood) from comparing linear mixed-effects models with *versus* without the fixed effect of interest. A significant drop in deviance - determined by the likelihood ratio *χ*^2^ statistics - indicates that incorporating the fixed effect improves model fitting and explains more variance in the response variable than when it is removed from the model [40]. For each fixed effect, 30 sets of likelihood ratio *χ*^2^ and *p* values were generated; its significance in predicting the response variable was accepted when *all p* values passed *α* = 0.05, indicating that the effect was not biased by any influential point. Linear mixed-effects models were conducted in R using the lme4 package [40].

## 3 Results

### 3.1 Light modulates recognition memory performance

**Fig. 2:**
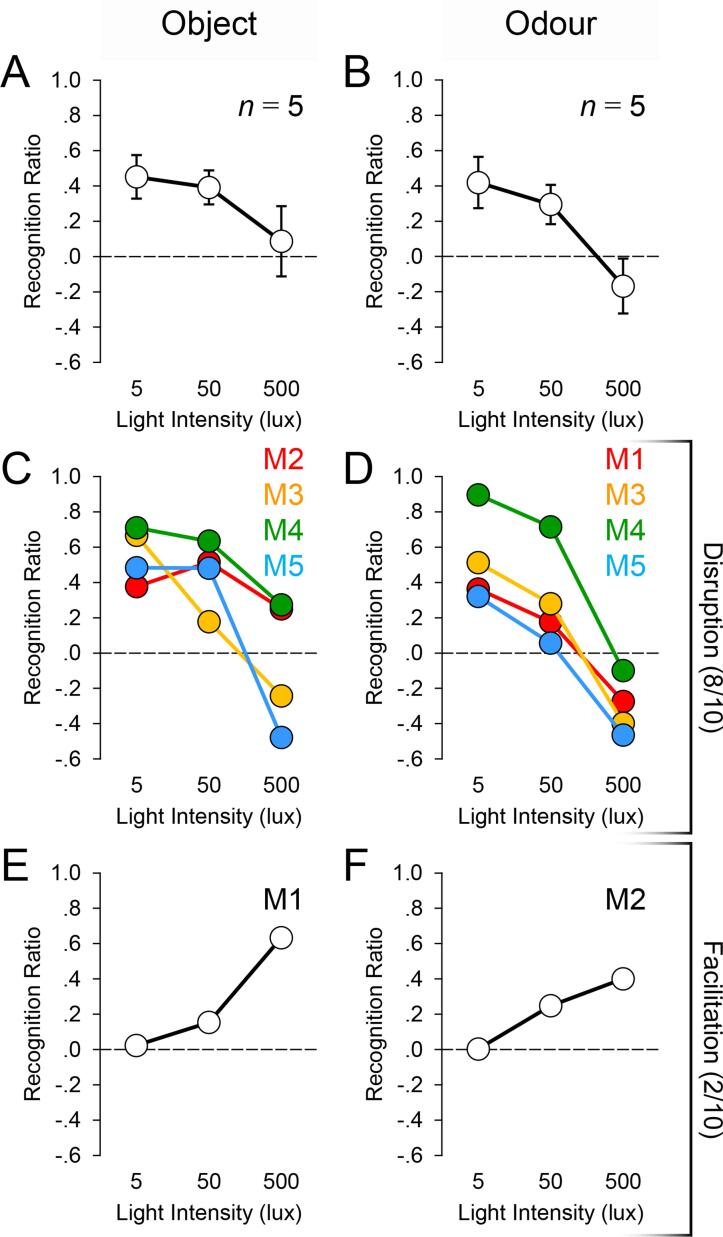
Light modulates recognition memory performance. Recognition memory performance on object and odour trials under 5-lux, 50-lux, and 500-lux lighting conditions in the 5 implanted mice. Panels A and B show the light-intensity-dependent disruptive effects on object and odour recognition memory performance at the group level. Panels C and D show that in 8 out of the 10 cases, light exerted intensity-dependent disruptive effects on performance at the individual level. Panels E and F show that in the remaining 2 cases, light exerted intensity-dependent facilitative effects on performance.

Object and odour recognition ratio scores, [(*New* − *Familiar*)/(*New* + *Familiar*)], from the 5 implanted mice under the different light levels are presented in Fig. 2A and 2B. Novelty preference is used as an indirect measure of recognition memory performance [27], [28], [29], [30]: *ratio* > 0 indicates a preference for the novel stimulus; *ratio* = 0 indicates no discrimination between novel and familiar stimuli; and *ratio* < 0 indicates a preference for the familiar stimulus.

There was a light-intensity-dependent disruptive effect on recognition memory performance, as confirmed by a Light Intensity (5, 50, or 500 lx) × Stimulus Type (Objects or Odours) within-subjects ANOVA. It showed a main effect of Light Intensity [*F*(2,8) = 4.725, *p* = 0.044]: the brighter the light level, the poorer the recognition memory performance. There was also a main effect of Stimulus Type [*F*(1,4) = 16.169, *p* = 0.016], due to overall better performance in the object task than in the odour task. However, there was no Light Intensity × Stimulus Type interaction [*F*(2,8) = 0.438, *p* = 0.660], suggesting that the disruptive effects of bright light on performance were comparable for the two types of stimulus. Consistent with these results from the ANOVA, one-sample *t* tests found that, when pooled across object and odour trials, mean recognition scores in the 5-lux and 50-lux conditions were both significantly higher than the value of 0 [*t*(4) = 3.682, *p* = 0.021 (2-tailed) and *t*(4) = 3.815, *p* = 0.019 (2-tailed), respectively], but the mean recognition score in the 500-lux condition was not different from 0 [*t*(4) = −0.266, *p* = 0.803], further confirming that exposure to the 500-lux light pulse, but not 5-lux and 50-lux light pulses, disrupted recognition memory performance. The impaired performance under the 500-lux condition was not driven by any change in general activity, because there was no significant main effect of Light Intensity on sample exploration or distance travelled: *exploration* = 11.40 ± 1.37 s min^−1^, 12.06 ± 1.48 s min^−1^, and 12.87 ± 1.45 s min^−1^ under 5, 50, and 500 lx, respectively; *distance travelled* = 0.79 ± 0.09 m min^−1^, 0.81 ± 0.10 m min^−1^, and 0.88 ± 0.07 m min^−1^ under 5, 50, and 500 lx, respectively (*p*s > 0.6).

### 3.2 Variability in response to light

#### 3.2.1 Implanted animals

Although group-level data of the 5 implanted mice showed a light-intensity dependent disruption on performance, a closer examination of individual-level data indicated that the opposite pattern was found on 2 out of the 10 occasions. On object trials, mice M2, M3, M4, and M5 showed their worst performance under 500 lx (Fig. 2C), but mouse M1 showed its best performance at this light level (Fig. 2E). Similarly, on odour trials mice M1, M3, M4, and M5 showed their best performance at 5 lx (Fig. 2D), but mouse M2 showed its worst odour performance at this light level (Fig. 2F). Thus, brighter light can also have a *facilitative* effect on the mouse’s recognition performance in some cases.

#### 3.2.2 Non-implanted animals

The variability in the effect of light on performance could be due to implantation-related side effects (e.g., implant weight interfering with exploration under certain conditions). Although unlikely, we attempted to rule out this possibility by replicating the experiment with non-implanted mice. Twelve non-implanted mice received one object recognition trial under 10 lx and one object trial under 350 lx in a counterbalanced order. Similar to the 5 implanted mice, group-level data of the 12 non-implanted mice showed a light-intensity dependent disruption, with an average recognition ratio of 0.373 ± 0.044 under 10 lx and an average ratio of 0.108 ± 0.113 under 350 lx [*F*(1,11) = 5.205, *p* = 0.043]. The mean ratio under 10 lx was significantly different from 0 [one-sample *t*(11) = 8.544, *p* < 0.001] but the mean ratio under 350 lx was not [one-sample *t*(11) = 0.959, *p* = 0.358]. Inspection of individual-level data from these non-implanted mice showed that, in 9 out of the 12 mice, there was a light-intensity-dependent disruptive effect; mean recognition ratios from these 9 cases were 0.396 ± 0.041 under 10 lx *versus* − 0.046 ± 0.079 under 350 lx. However, in the remaining 3 cases there was a light-intensity-dependent facilitative effect; mean recognition ratios from these 3 cases were 0.301 ± 0.053 under 10 lx *versus* 0.572 ± 0.096 under 350 lx. The differential effects of light on performance in these two subgroups were confirmed by a Light Intensity (10 *versus* 350 lx) × Subgroup (Disrupted *versus* Facilitated) interaction [*F*(1,10) = 18.403, *p* = 0.002], due to impaired performance in the Disrupted subgroup and improved performance in the Facilitated subgroups under the brighter light [Disrupted *versus* Facilitated under 350 lx =  − 0.046 ± 0.079 *versus* 0.572 ± 0.096; simple effect of Subgroup: *F*(1,10) = 10.400, *p* = 0.009]. In contrast to recognition memory performance, object exploratory activity was comparable under the two lighting conditions and between the two subgroups [main effect of Light Intensity: *F*(1,10) = 0.047, *p* = 0.833; main effect of Subgroup: *F*(1,10) = 0.871, *p* = 0.373; Light Intensity × Subgroup interaction: *F*(1,10) = 0.023, *p* = 0.882].

**Fig. 3:**
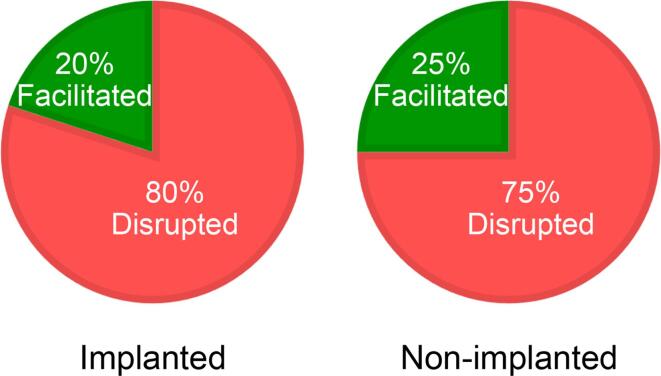
Variability in the effect of light on recognition memory performance. The *left* panel shows that, in the implanted mice, in 8 out of the 10 cases there was a light-intensity dependent disruption on performance (*red*); in the remaining 2 cases there was a light-intensity dependent facilitation (*green*). Similarly, the *right* panel shows that, in the non-implanted mice, in 9 out of the 12 cases there was a light-intensity dependent disruption (*red*); in the remaining 3 cases there was a light-intensity dependent facilitation (*green*).

Thus, variability in the effect of light on recognition memory performance can be found in both implanted and non-implanted mice: 75–80% of the cases showed a light-intensity-dependent disruption, whereas the remaining 20–25% of the cases showed a light-intensity-dependent facilitation (Fig. 3).

### 3.3 Light strengthens the relationship between EEG *θ*/*γ* power and recognition memory performance

**Fig. 4:**
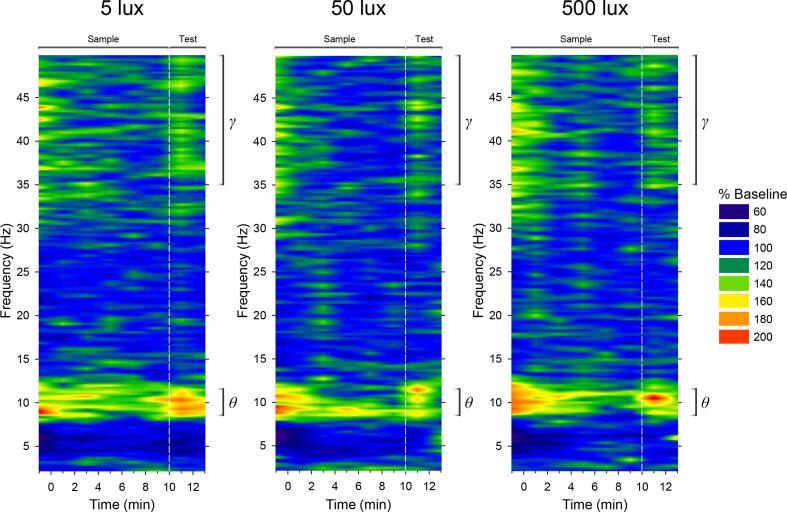
Normalised EEG spectra under different light intensities. Spectral heatmaps show normalised and group-averaged EEG time–frequency spectra from object recognition trials under 5 lx (*left* panel), 50 lx (*middle* panel), and 500 lx (*right* panel). EEG spectra (in μV^2^ per 0.25 Hz) during sample and test phases were normalised to the EEG spectrum (in μV^2^ per 0.25 Hz) in the corresponding frequency bins during the baseline period. Fast *θ* (8.5–11.2 Hz) and slow *γ* frequency bands (35–50 Hz) are indicated in each panel. The warmer the colour (*red*) is on the heatmap, the greater is the increase in EEG power at a particular frequency relative to baseline; whereas darker colours (*blue*) indicate that there is minimal change or a decrease in EEG power at a particular frequency relative to baseline.

Could the variability in performance be partly related to brain activity, and if so, did light alter the relationship between brain activity and performance? These questions are examined using the EEG data from the 5 implanted animals. We first checked that basal *θ* and *γ* power in μV^2^ during the baseline period preceding light exposure did not differ between conditions (main effects of Light Intensity: *p*s > 0.8; Table 1) and did not bear any relationship to subsequent exploratory activity or performance (linear mixed-effects models: *p*s > 0.2). We then expressed EEG power spectra during sample and test phases as a percentage of the EEG spectrum in the corresponding frequency bins during the baseline period; examples of normalised EEG spectra under different lighting conditions are shown in Fig. 4. Linear mixed-effects models [40] were conducted with: recognition ratios, stimulus exploration times, or distance travelled as the response variable; and *θ*/*γ* Power and Light Intensity × *θ*/*γ* Power as fixed effects. Effects of *θ*/*γ* power were statistically indistinguishable between object and odour trials, as indicated by nonsignificant Stimulus Type × *θ*/*γ* Power interaction terms in linear mixed-effects models; hence Stimulus Type and its interaction terms were not entered as fixed effects in subsequent linear mixed-effects models.

**Table 1:** Mean (±Standard Error of the Mean) Absolute *θ* and *γ* Values (in μV^2^ per 0.25 Hz) Prior to Normalisation and Novel and Familiar Stimulus Exploration Times (in seconds) in Implanted Mice.

Trial	lux	*θ*_B_	*γ*_B_	*θ*_S_	*γ*_S_	*θ*_T_	*γ*_T_	*E*_N_	*E*_F_
	5	17.421 (2.229)	5.922 (0.988)	23.601 (3.610)	6.284 (0.998)	26.488 (1.588)	6.737 (0.926)	36.20 (5.919)	12.32 (1.670)
Object	50	17.381 (2.692)	5.593 (1.180)	24.500 (1.616)	5.940 (0.979)	22.941 (3.021)	5.825 (1.137)	39.42 (9.961)	14.50 (0.823)
	500	17.671 (2.294)	5.690 (0.958)	23.689 (1.566)	6.489 (0.939)	23.655 (2.002)	6.178 (0.992)	27.50 (6.175)	21.80 (4.379)
	5	15.865 (2.144)	5.310 (1.170)	19.039 (2.965)	5.516 (1.213)	21.472 (2.088)	5.155 (0.914)	23.52 (2.018)	10.20 (2.938)
Odour	50	16.413 (2.115)	5.435 (1.116)	20.732 (2.960)	5.602 (1.109)	20.195 (1.867)	5.548 (0.959)	21.02 (5.345)	10.24 (1.780)
	500	16.557 (2.264)	5.291 (1.131)	22.448 (3.422)	5.291 (1.057)	23.145 (3.699)	5.346 (0.986)	16.06 (4.338)	20.96 (2.556)

*θ*_B_, baseline *θ*; *γ*_B_, baseline *γ*; *θ*_S_, sample *θ*; *γ*_S_, sample *γ*; *θ*_T_, test *θ*; *γ*_T_, test *γ*; *E*_N_, novel stimulus exploration times at test; *E*_F_, familiar stimulus exploration times at test.

#### 3.3.1 EEG θ power

**Fig. 5:**
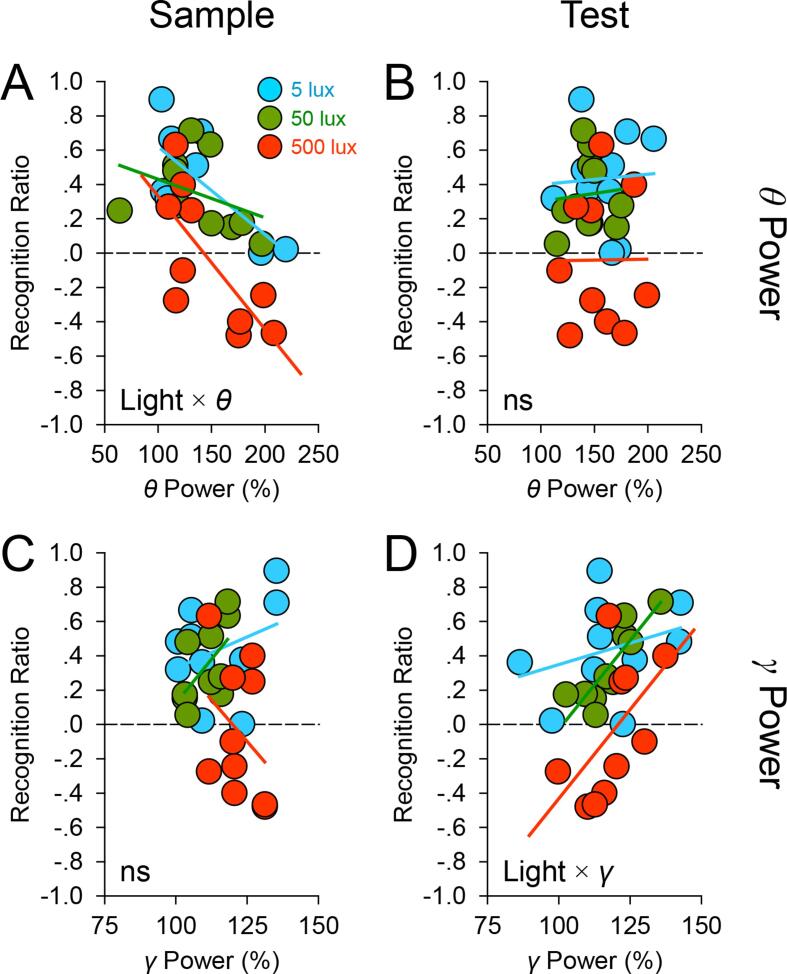
Light strengthens the relationship between EEG *θ*/*γ* power and performance. Scatter plots show the relationships between *θ*/*γ* power and recognition performance under the different lighting conditions (*blue*, 5 lx; *green*, 50 lx; and *red*, 500 lx; *coloured lines*, least-square linear regression fits) in the 5 implanted mice. EEG spectra (in μV^2^ per 0.25 Hz) during sample and test phases were expressed as a percentage of the EEG spectrum (in μV^2^ per 0.25 Hz) in the corresponding frequency bins during the baseline period. In each panel, there are 30 data points (5 mice × 2 types of stimulus × 3 light intensities). Panel A shows strengthening of the effect of sample EEG *θ* power on recognition memory performance when light intensity was increased from 5 and 50 lx to 500 lx (Light × *θ* Power interaction in the linear mixed-effects model); panel B shows the statistically nonsignificant effect of test *θ* power on performance; panel C shows the nonsignificant effect of sample *γ* power on performance; and panel D shows strengthening of the effect of test *γ* power on performance when light intensity was increased from 5 lx to 50 and 500 lx (Light × *γ* Power interaction in the linear mixed-effects model).

**Fig. 6:**
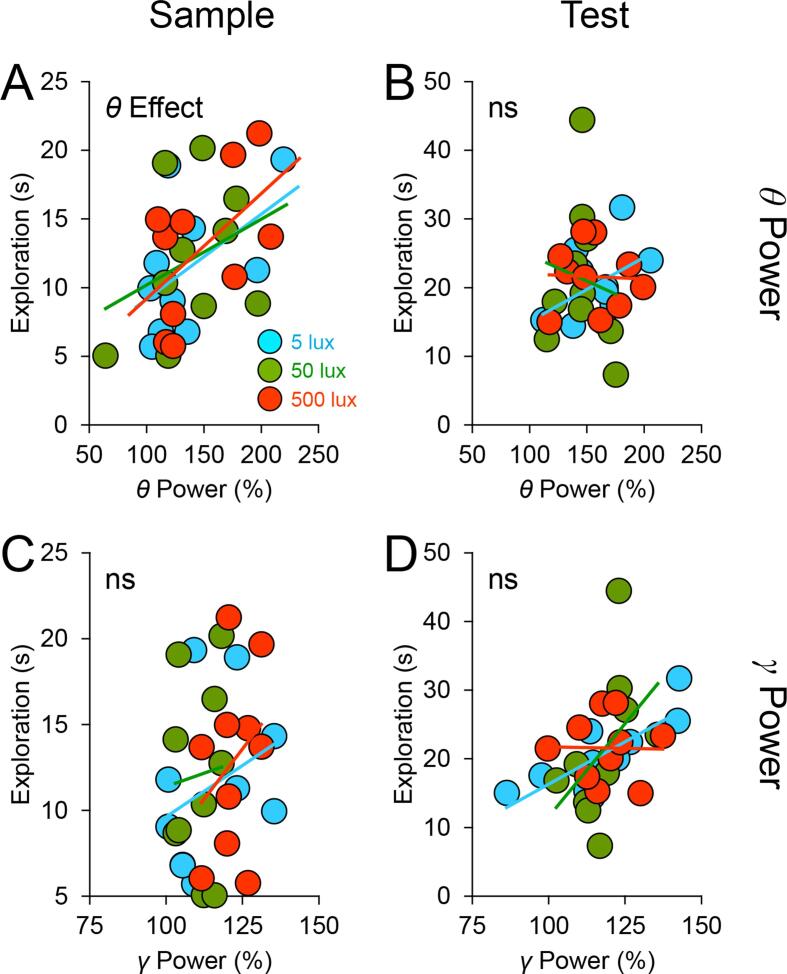
The relationship between *θ* power and exploration is unaffected by light. Scatter plots show the relationships between *θ*/*γ* power and stimulus exploration (s min^−1^) under the different lighting conditions (*blue*, 5 lx; *green*, 50 lx; and *red*, 500 lx; *coloured lines*, least-square linear regression fits) in the 5 implanted mice. EEG spectra (in μV^2^ per 0.25 Hz) during sample and test phases were expressed as a percentage of the EEG spectrum (in μV^2^ per 0.25 Hz) in the corresponding frequency bins during the baseline period. Panel A shows that effects of sample *θ* power on stimulus exploration in the sample phase were comparable across the different lighting conditions (i.e. no Light × *θ* Power interaction in the linear mixed-effects model); panels B-C show statistically nonsignificant effects of test *θ*, sample *γ*, and test *γ* power on stimulus exploration.

Theta power during the sample phase was negatively related to performance (likelihood ratio *χ*^2^ ≥ 11.157, *p* ≤ 0.0008; Fig. 5A). The Light Intensity × Sample *θ* Power interaction term was also significant (likelihood ratio *χ*^2^ ≥ 8.219, *p* ≤ 0.016), indicating that the association between sample *θ* power and performance was enhanced by increasing light intensity (Fig. 5A). In addition, sample *θ* power was positively related to sample exploration (likelihood ratio *χ*^2^ ≥ 8.795, *p* ≤ 0.003; Fig. 6A). However, the association between sample *θ* power and sample exploration remained unchanged under the different lighting conditions (*p* ≥ 0.455; Fig. 6A). Similarly, there was some indication of a positive relationship between sample *θ* power and distance travelled, although it failed to pass the jackknife criterion (0.005 ≤ *p* ≤ 0.064); this was unaffected by increasing light intensity (*p* ≥ 0.293). In contrast to the significance of sample *θ* power in predicting performance and general activity, test *θ* power was unrelated to these response variables (Fig. 5B and 6B).

#### 3.3.2 EEG γ power

A different pattern of results was found for higher frequency brain signals. Sample *γ* power was unrelated to performance (*p* ≥ 0.155; Fig. 5C); whereas a positive relationship was found between test *γ* power and performance (likelihood ratio *χ*^2^ ≥ 3.086, *p* ≤ 0.051; Fig. 5D). The Light Intensity × Test *γ* Power interaction term was also significant (likelihood ratio *χ*^2^ ≥ 6.206, *p* ≤ 0.045), indicating that the association between test *γ* power and performance was strengthened by brighter light (Fig. 5D). Associations between *γ* power and general activity did not pass the jackknife criterion (sample *γ* power and sample exploration: 0.035 ≤ *p* ≤ 0.124, Fig. 6C; test *γ* power and test exploration: 0.010 ≤ *p* ≤ 0.093, Fig. 6D; sample *γ* power and distance travelled: 0.006 ≤ *p* ≤ 0.453).

#### 3.3.3 Summary

Thus, EEG *θ* and *γ* band signals predict recognition memory performance, and these relationships between *θ*/*γ* signals and performance are strengthened by increasing light intensity. EEG *θ* power is also related to exploratory activity, but this relationship is unaffected by light. These results are summarised in Table 2.

**Table 2:** A Summary of Fixed Effects from Linear Mixed-effects Models.

Response Variable	Fixed Effect*	Significance†
Recognition ratio	Sample *θ* Power	yes
	Sample *γ* Power	no
	Test *θ* Power	no
	Test *γ* Power	yes
	Light × Sample *θ* Power	yes
	Light × Sample *γ* Power	no
	Light × Test *θ* Power	no
	Light × Test *γ* Power	yes

Stimulus exploration (s min^−1^)	Sample *θ* Power	yes
	Sample *γ* Power	no
	Test *θ* Power	no
	Test *γ* Power	no
	Light × Sample *θ* Power	no
	Light × Sample *γ* Power	no
	Light × Test *θ* Power	no
	Light × Test *γ* Power	no

Distance travelled (m min^−1^)	Sample *θ* Power	no
	Sample *γ* Power	no
	Light × Sample *θ* Power	no
	Light × Sample *γ* Power	no

* EEG *θ*/*γ* power was normalised to basal *θ*/*γ* power.

† Significance of the fixed effect was determined by examining the change in deviance (−2 × maximum log-likelihood) from comparing linear mixed-effects models with *versus* without the fixed effect of interest. A significant drop in deviance – determined by the likelihood ratio *χ*^2^ statistics – indicates that incorporating the fixed effect improves model fitting and explains more variance in the response variable than when it is removed from the model [40].

## 4 Discussion

In humans, light can affect alertness, brain activity, and cognitive performance [41], [42], [12], [13], [14], [15], [16]. Although mice are nocturnal, their response to light across the circadian cycle - as described by the phase-response curve (PRC) - is broadly similar to that in humans [17]. Here we assessed the effects of different light intensities (5, 50, and 500 lx) on waking EEG and cognitive performance in C57/BL6 mice, providing a model for light-alerting effects in humans.

### 4.1 Light as a modulator of arousal and performance

In mice, light exerted an intensity-dependent effect on recognition memory performance; in 75–80% of the cases, the brighter the light, the poorer the performance; whereas in the remaining 20–25% of the cases, the brighter the light, the better the performance. In addition, by using different light intensities to modulate physiological arousal [18], [19] we identified waking EEG correlates of recognition memory performance. EEG *θ* signals in the sample phase negatively predicted recognition memory performance, whereas *γ* signals in the test phase positively predicted performance, and these associations were strengthened by increasing light intensity. Effects of EEG *θ*/*γ* signals on recognition memory performance were not driven by changes in stimulus exploration.

The relationship between light-modulated *θ* signals and task performance is comparable between humans and mice. In humans, bright light leads to decreased waking EEG *θ*/*α* and slow-wave *δ* activity associated with sleep pressure [4], [5], [6], [7], [8], [9], [10], [11]. These light-modulated EEG spectral changes reduce sleepiness, and in some cases, enhance reaction-time performance [42]. Thus, despite many major procedural discrepancies between human studies and our current mouse study, the finding is broadly similar: the strength of light-modulated *θ* signals is negatively related to task performance in both species.

Although *θ* and *γ* signals were not directly measured from the hippocampus in our study, hippocampal local field potentials are detectable at the cortical level in mice [38], [43] due to their relatively thin cortex of ~1 mm [44]. In this regard, our results are consistent with the well-documented finding that hippocampal *θ* activity is a reliable predictor of behavioural performance in rodents [45], [46], [47], [48], [49], [50], [51], [52], [53], [54], [55]. There are two types of *θ* activity in the rodent brain [56], [57]: type 1 (7–12 Hz) is related to locomotion and exploration and is more prominent in the septal pole along the septotemporal axis of the hippocampus [58]; whereas type 2 (4–9 Hz) is related to emotional processing and heightened arousal and can be elicited by activating cells in the temporal pole of the hippocampus [59]. In our study, the positive association between *θ* power and exploration (Fig. 6A) could be of type 1 origin [58], but the strengthening of the negative association between *θ* power and novelty preference with increasing light intensity (Fig. 5A) may involve type 2-related arousal [56], [59]. Whilst the exact origin and type of *θ* activity in our study remain to be determined, the light-modulated *θ* signal is likely to reflect heightened network activity in the septohippocampal system [60], [61] due to increased attentional processing of the environment [62], [63], [64], enhancing investigation of the familiar stimulus at the expense of the novel stimulus. Furthermore, our results are consistent with the notion that slow *γ* band activity in the hippocampus and interconnected cortical areas is crucial for mnemonic processes [65], [66]. In our experiment, the light-modulated *γ* signal could promote the retrieval of stored object and odour representations [34]. Together with our previous reports, we suggest that light modulates physiological arousal [18] and *θ* and *γ* band brain activities involved in attentional and mnemonic processes, thereby affecting recognition memory performance [26].

### 4.2 Potential role of respiration-related rhythms

In recent years there have been numerous studies examining respiration-related rhythms, which can be detected from, as well as entrain, local field potentials in different parts of the rodent brain [67], [68], [69]. For example, whisking and sniffing can generate rhythmic signals ranged from ~4 Hz during quiet wakefulness to >10 Hz during object exploration [70] and odour exploration [71], which coincides with the fast *θ* frequency band in our study. These respiration rhythms originate from ascending olfactory regions, propagating to different downstream areas [72]. On the other hand, *θ* activity is traditionally thought to originate from *θ*-burst firing of neurons in the medial septum, as damage to this area abolishes all *θ* activity in the cortex [73] and hippocampus [74]. Thus, respiration rhythms and *θ* activity have distinct origins but overlapping frequency ranges. In addition, a recent study suggests that changes in *θ* activity may precede and drive changes in respiration rhythms [69]. Without monitoring respiratory activity (e.g., using a nasal thermocouple), it is not possible to distinguish olfactory-driven respiration rhythms from septohippocampal-driven *θ* activity. Both types of rhythmic activity could potentially contribute to the positive relationship between EEG power in the 8–11 Hz band and stimulus exploration (Fig. 6A), and this remains to be examined in future studies.

### 4.3 Potential sources of variability in response to light

It is unclear why the light-intensity-dependent effect on performance was reversed in some cases. In humans, there are individual differences in response to light [15], [75]. For example, in a recent human study examining melatonin suppression in response to evening light, it was reported that there was variability in participants’ sensitivity to light, with a >50-fold difference between the most-sensitive and least-sensitive participants. The group-averaged effective light level for half-maximal melatonin suppression, termed EC_50_, was at ~25 lx, but individual EC_50_ values could range from under 10 lx to ~350 lx [76]. Photic response variability in humans comes from at least two sources of variation: (*a*) the exact time of the internal clock at which light is presented; and (*b*) homeostatic sleep pressure [15].

#### 4.3.1 Phase response curve and circadian period

Our previous mouse studies examining effects of light on electrocardiogram (ECG) and sleep may also shed some light on this matter: a bright light of ~700–1000 lx elevated the mouse’s heart rate at subjective midday (CT6) [18], but it supressed heart rate, reduced body temperature, and induced sleep at night [18], [36]. This suggests that the same light intensity can exert opposite effects on physiological arousal depending on the time of the internal clock.

In fact, interindividual and intraindividual variability in light sensitivity is also well documented in mice. Notably, the size of a light-induced phase shift is known to be correlated with the mouse’s endogenous period, *τ*: the faster the internal clock free runs, the greater is the phase delay [77]. Putting it in the context of the current experiment, it can be assumed that there is between-subjects as well as trial-to-trial variability in *τ* when mice are released into DD at the beginning of the day. The internal clock could free run faster or slower, depending on the actual *τ* of the mouse/day. When the internal clock free runs at the average endogenous speed (i.e. *τ* slightly less than 24 h), the mouse will receive recognition testing and the light pulse near subjective midday (CT6), a time when light sensitivity is low; this is indicated by the “*dead zone*” on the phase response curve (PRC) of the mouse [77]. By contrast, when the internal clock free runs at a faster speed (due to shortened *τ*), the light pulse will be presented at the later portion of the subjective day, a time when light sensitivity starts to increase [77]. In addition, our lighting protocol - which involved (unintended) intermittent exposure to short-day photoperiods (6 h:18 h LD) - may have accumulating effects, gradually destabilising the circadian clock [78] and exacerbating the variability in *τ* and clock speed [77].

Based on this account, the reversed light-intensity-dependent effect in 2 out of the 10 cases could be partly due to the internal clock free running at a speed faster than the average speed, and as a result, light pulses hit the *later*, more sensitive part of the PRC. In fact, variability in clock speed - which depends on dopamine levels - is a feature of internal timing [79], and it could be driven by dopamine-dependent ultradian rhythms of spontaneous activity [80].

#### 4.3.2 Sleep pressure and circadian photic sensitivity

Another related source of variability in light sensitivity may come from trial-to-trial variation in the behavioural state preceding each trial [15], [81]. In the current experiment, basal *θ* and *γ* power during the baseline period preceding light exposure did not differ between lighting conditions and did not bear any relationship to subsequent recognition memory performance. Nevertheless, the mouse’s sleep pressure at midday is much lower than that at the beginning of the day, and there will be some variation in the vigilance state prior to the baseline period. Of relevance to our results is the fact that adenosine-dependent increases in sleep pressure can reduce the photic responsiveness of the clock located in the suprachiasmatic nucleus (SCN) [82]. In the context of our current experiment, if the mouse has been awake for a certain period of time prior to the trial, some sleep pressure will be accumulated, and thus the SCN will be less sensitive to light. But if the mouse has consolidated sleep prior to the trial, sleep pressure will be minimal, and the SCN will be more sensitive to light; as a result, downstream brain regions that receive monosynaptic and multisynaptic projections from the SCN will be more affected by light.

#### 4.3.3 The role of the SCN

These accounts rely on the fact that the SCN - which is densely innervated by melanopsin-expressing photosensitive retinal ganglion cell (pRGC) axon terminals - is responsible for mediating the acute effect of light on performance and the variability in these responses. In fact, both facilitative and disruptive effects of light on performance require pRGCs [23], [26]. In addition, it has recently been reported that distinct projections from different subpopulations of pRGCs to the SCN and habenula region mediate long-term effects of light on recognition memory performance *versus* mood-related behaviour [21]. Further studies are needed to confirm the role of the SCN (and other retinorecipient regions) in mediating the acute effect of light on performance and the variability in these responses.

## 5 Conclusion

Light can modulate physiological arousal, brain activity, and cognitive performance. This has translational implications for the use of light therapy in many disorders; a recent example is that light-modulated slow *γ* activity in the cortex and hippocampus can have neuroprotective effects in mouse models of neurodegenerative [83] and neurovascular diseases [84]. However, our results suggest that whether light has an intensity-dependent disruptive or facilitative effect on recognition memory performance may partly depend on other extraneous factors, such as the exact phase of the internal clock at which light is presented and preceding vigilance state. Future studies are needed to clarify the relationships among *τ*, light sensitivity, and preceding behavioural state, and how these factors are translated into performance.

## Author contributions

Conceptualization: SH, SKET, VVV, DMB, SNP; data curation: SH; formal analysis: SH, SKET, VVV, SNP; funding acquisition: RGF, SNP; investigation: SH; methodology: SH; project administration: SH, SNP; resources: SH, RGF, SNP; software: SH, VVV; supervision: VVV, DMB, SNP; validation: SH, VVV; visualization: SH, SKET; writing - original draft: SH, SKET, RGF, VVV, DMB, SNP; writing - review & editing: SH, SKET, RGF, VVV, DMB, SNP.

## Declaration of Competing Interest

The authors declare that they have no known competing financial interests or personal relationships that could have appeared to influence the work reported in this paper.
